# A single entomopathogenic nematode infection assay for *Drosophila melanogaster* larvae

**DOI:** 10.1016/j.mex.2025.103157

**Published:** 2025-01-06

**Authors:** Sreeradha Mallick, Eric Kenney, Jacob Rashap, Ioannis Eleftherianos

**Affiliations:** Infection and Innate Immunity Lab, Department of Biological Sciences, The George Washington University, Washington, DC 20052, USA

**Keywords:** Host-parasite interaction, Anti-nematode immunity, Nematode pathogenesis, Nematode infection assay

## Abstract

The entomopathogenic nematodes (EPNs) Steinernema carpocapsae and Steinernema hermaphroditum can efficiently infect the fruit fly, Drosophila melanogaster. The EPN infective juvenile (IJ) stage is the free-living and non-feeding stage that seeks out suitable insects to infect. While previous studies have described successful infection of *D.* melanogaster larvae with a standard amount of 100 IJs, the pathogenicity of a single IJ nematode towards insects remains poorly understood. Given the variability in pathogenesis among individual IJs, investigating the ability of a single IJ to cause infection in fly larvae addresses a significant gap in our understanding of host-parasite interactions. This protocol aims to evaluate the infection efficiency of a single IJ of S. carpocapsae and S. hermaphroditum. This information contributes towards a better understanding of the EPN-insect interactions and promises to improve the use of EPNs in pest management strategies. The method is summarized below:

• Individual third instar wild-type *D. melanogaster* larvae are infected with either a single IJ or 100 IJs of either nematode species. Uninfected control larvae are treated with sterile water.
• Larval survival is monitored over time.
• Survival curves are generated, and results are statistically analyzed to determine the effect of a single nematode on the fly larval mortality.

## Graphical abstract

**Figure:**
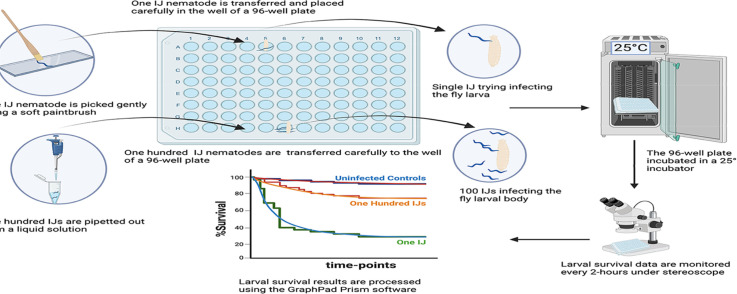

Specifications table

**Table:** 

Subject area:	Immunology and Microbiology
More specific subject area:	Parasitology
Name of your method:	Nematode infection assay
Name and reference of original method:	N/A
Resource availability:	N/A

## Background

*Steinernema* are entomopathogenic (EPN) nematodes that infect and kill insects [1]. They share a mutualistic relationship with *Xenorhabdus* bacteria, which provide them with essential nutrients and also guard them from environmental stressors [2]. Upon infection, the symbiotic bacteria produce toxins and metabolites that facilitate the killing of the host insect [3]. The life cycle of *Steinernema* nematodes begins when the infective juvenile (IJ) stage, which is the free-living and non-feeding stage, enters an insect host by penetrating its natural orifices and migrating into the hemocoel [4]. The IJ is a developmentally arrested stage until the parasitic nematode finds a suitable host [5,6].

In *Steinernema carpocapsae*, the third-stage larva becomes the IJs [7]. This EPN is mutualistically associated with the bacteria *Xenorhabdus nematophila* [8]. When the *S. carpocapsae* IJs kill the insect, they continue their development and mating, which leads to multiple generations in the host. When nutrients in the infected insect decline, new *S. carpocapsae* IJs develop and emerge from the host carcass [9]. *Steinernema hermaphroditum* is another EPN with a unique life cycle, where hermaphroditic individuals are produced in the first generation, and males and females appear in the second generation [10]. *Steinernema hermaphroditum* forms an obligate symbiotic relationship with the bacteria *Xenorhabdus griffiniae* and undergoes four larval developmental stages (J1-J4) before maturing as an adult. When the nematodes reach the second larval stage, they transition into the IJs [11].

*Drosophila melanogaster* is a well-established model for dissecting the molecular basis of the host innate immune response against various pathogens, including parasitic nematode [12]. Recent research on the survival of *D. melanogaster* larvae following infection by *Steinernema* nematodes has revealed that *S. carpocapsae* exhibits greater pathogenicity compared to *S. hermaphroditum*. The difference in pathogenicity between the two *Steinernema* species suggests that *S. carpocapsae* and symbionts *X. nematophila* use different infection strategies compared to *S. hermaphroditum*-*X. griffiniae* to infect *D. melanogaster* larvae [13].

Standard nematode infection assays involve exposure of *D. melanogaster* to 100 nematode IJs [14]. However, the ability to infect an insect host can vary substantially between individual IJs. In other words, not all IJs are effective in causing insect infection. The current method describes a simple single EPN infection protocol that tests the ability of single *S. carpocapsae* and *S. hermaphroditum* IJs to successfully infect and kill *D. melanogaster* larvae. Establishing this assay will contribute to improving the use of EPNs in biological control applications for the efficient management of insect pests and disease vectors.

## Method details

Three 96-well plates were prepared by filling each well with 100 µL of 1.25% agarose gel, which was allowed to cool for three hours. In all three plates, a single third instar *D. melanogaster* larva of the *w^1118^* strain was placed in each well. For the first plate, which served as the uninfected control treatment, 10 µL of sterile water only were added to each well. In the second plate, a single IJ of either *S. carpocapsae* or *S. hermaphroditum* nematodes was placed directly using a fine paintbrush into 10 µL of sterile water. In the third plate, 100 IJs of either *S. carpocapsae* or *S. hermaphroditum*, suspended in 10 µL of sterile water, were pipetted into each well. The wells were then sealed with Master clear real-time PCR films (Eppendorf, Enfield, CT), which were manually perforated to allow airflow. The plates were kept in an incubator at 25 °C under a 12:12 hour light/dark photoperiod, and larval survival was monitored at two hours intervals and over 96 h. Each experiment was replicated at least three times. Larval survival data were recorded and analyzed, and Kaplan-Meier survival curves were generated using the GraphPad Prism software and log-rank (Mantel–Cox) test was used to assess the statistical significance differences between the different experimental treatments.

## Method validation

**Fig. 1:**
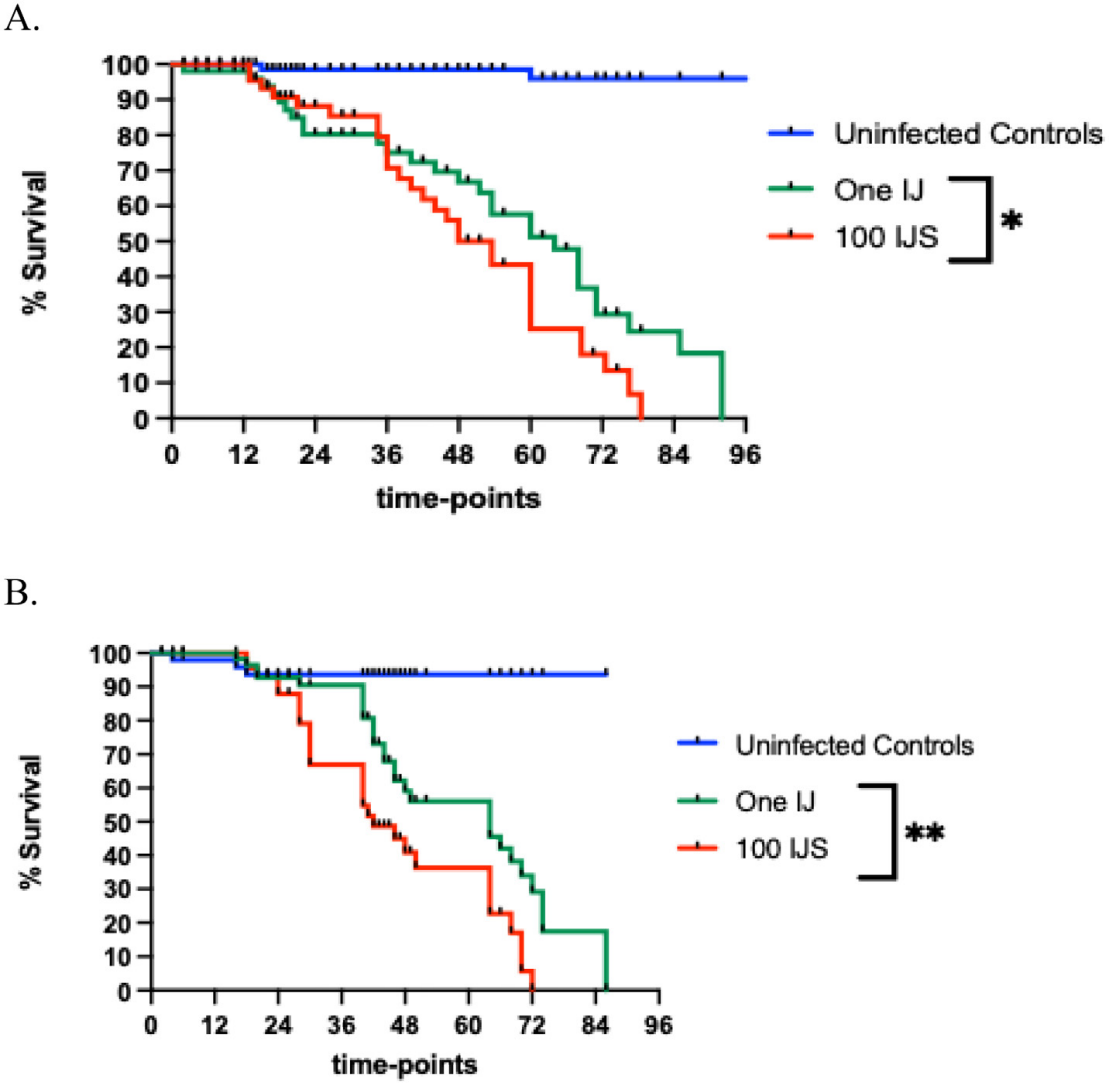
Effect of entomopathogenic nematode infection on the survival of *Drosophila melanogaster* third instar larvae. (A) Survival percentage of *w^1118^* strain *D. melanogaster* larvae over 96 h following infection with either one or 100 infective juveniles (IJs) of the entomopathogenic nematode *Steinernema carpocapsae*. The uninfected control larvae were treated with 10 µL of sterile water. Survival curves were compared using a Log-rank test, revealing a statistically significant difference between the single IJ and 100 IJs infection groups (✱ *P* = 0.0494). (B) Survival percentage of *w^1118^* strain *D. melanogaster* larvae over 96 h following infection with either one or 100 *Steinernema hermaphroditum* IJs. The uninfected control larvae were treated with 10 µL of sterile water. Survival curves were compared using a Log-rank test, showing a statistically significant difference between the single IJ and 100 IJ infection groups (✱✱ *P* = 0.0065). Survival data represent mean values from three independent experiments.

The survival trend of the *D. melanogaster* third instar *w^1118^* larvae following infection with *Steinernema* IJs reveals that infection with a single IJ can cause larval death. When *D. melanogaster* larvae were infected with a single *S. carpocapsae* IJ, 90% mortality was observed at 84 h. In contrast, exposure of *D. melanogaster* larvae to 100 *S. carpocapsae* IJs resulted in 90% mortality at 72 h (Fig. 1A). For *S. hermaphroditum*, a single IJ caused 90% larval mortality at 72 h. When 100 *S. hermaphroditum* IJs were used to infect the fly larvae, 90% mortality occurred at 60 h (Fig. 1B). These results suggest that single IJs from either *Stenernema* nematode species can produce infection factors to undermine the *D. melanogaster* immune response, which leads to larval death.

**Fig. 2:**
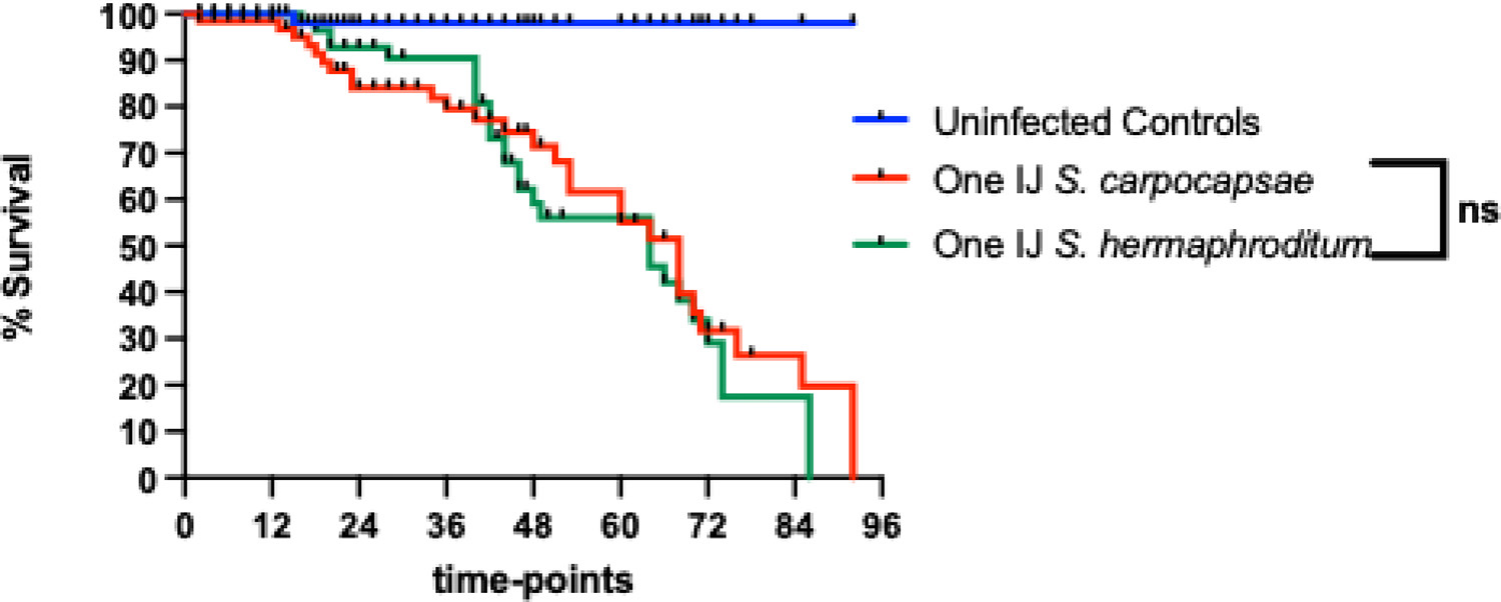
Comparison between the survival of third instar larvae of the *Drosophila melanogaster* strain *w^1118^* following infection with infective juveniles (IJs) of the entomopathogenic nematodes *Steinernema carpocapsae* and *S. hermaphroditum*. Larval survival was monitored over a course of 96 h after exposure to a single IJ of either nematode species. Survival curves were compared using a Log-rank test in GraphPad Prism. There was no significant (ns) statistical difference in the survival rates between the two infection groups.

Comparison between the survival curves of *D. melanogaster* third instar *w^1118^* larvae infected with a single IJ of either *S. carpocapsae* or *S. hermaphroditum* shows that the latter EPN species is slightly more pathogenic than the former. More precisely, single IJs of *S. carpocapsae* kill *D. melanogaster* larvae approximately 10 h faster than single IJs of *S. hermaphroditum* nematodes. However, no statistically significant differences were found between the survival curves of the two nematode infection groups (Fig. 2).

## Conclusions

The current single EPN infection protocol allows us to estimate the infection ability of single *Steinernema* IJs against *D. melanogaster* wild type larvae. Results from validating this method confirm and expand our previously findings that *Steinernema* nematodes produce effector molecules to disarm the fly immune system and defeat the host [15,16]. The same experimental approach can be used to investigate the interaction between EPNs and *D. melanogaster* mutants to test the role of host immune genes with putative anti-nematode immune properties [17]. Following previous advances on generating tools for the genetic manipulation of EPNs through the development of an RNAi-mediated gene knockdown technique [18], the current infection protocol will further promote research on understanding the molecular and functional basis of EPN parasitism.

## Limitations

1. *Larval Health*: It is crucial to ensure that the *D. melanogaster* larvae are healthy before the experiment. If the larvae in the stock culture are compromised, the nematode infection experiments will lead to misleading results in terms of larval survival. Fly survival experiments require proper maintenance of the fly stock culture under optimal conditions. Also, transferring the fly larvae with a fine paintbrush is critical to avoid wounding. Uninfected control larvae, in particular, are essential for establishing standard larval survival, which would allow proper comparisons between the uninfected and the infected larval groups.
2. *Transfer of IJs*: The IJs are delicate and require careful handling. If the IJs are damaged during transferring, the infection experiment will be compromised. Healthy IJs are usually slender and elongated under the microscope, while damaged IJs look distorted in shape. Also, injured IJs will not show any movement toward the fly larvae. Using a soft paintbrush and light pressure, a single IJ can be transferred gently from the glass slide into the well of the 96-well plates. This approach minimizes the risk of damaging the IJ while transferring and ensures that the IJ is motile and active for infection.
3. *Deposition of IJs*: The EPN IJs should be placed in close proximity to the *D. melanogaster* larvae to ensure efficient infection. This requires that the IJs are carefully deposited using a fine paintbrush near the fly larvae to facilitate nematode invasion and penetration.
4. *Maintaining Optimal Infection Conditions*: Single IJs and 100 IJ should be suspended in a 10 µL volume of sterile water, which is pipetted to each well of the 96-well plate. The volume of water in the well should be adequate to allow a single IJ to move freely and search for the *D. melanogaster* larva. It will also prevent the IJ from desiccation and provide them with optimal conditions to infect the fly larvae efficiently.

## Ethics statements

We have used fruit flies and entomopathogenic nematodes for our experiments, all of which complied with our institution's safety and laboratory guidelines.

## CRediT authorship contribution statement

**Sreeradha Mallick:** Conceptualization, Methodology, Investigation, Validation, Visualization, Writing – original draft, Writing – review & editing. **Eric Kenney:** Conceptualization, Methodology, Investigation, Validation, Visualization, Writing – review & editing. **Jacob Rashap:** Methodology, Investigation, Validation, Visualization, Writing – review & editing. **Ioannis Eleftherianos:** Supervision, Funding acquisition, Project administration, Resources, Conceptualization, Writing – review & editing.

## Declaration of competing interest

The authors declare that they have no known competing financial interests or personal relationships that could have appeared to influence the work reported in this paper.

## Data Availability

Data will be made available on request.
